# Green rebranding: Regenerative agriculture, future‐pasts, and the naturalisation of livestock

**DOI:** 10.1111/tran.12555

**Published:** 2022-07-08

**Authors:** George Cusworth, Jamie Lorimer, Jeremy Brice, Tara Garnett

**Affiliations:** ^1^ Oxford Martin Programme on the Future of Food, Oxford Martin School University of Oxford Oxford UK; ^2^ Hertford College University of Oxford Oxford UK

## Abstract

Anxieties around the relationship between livestock agriculture and the environmental crisis are driving sustained discussions about the place of beef and dairy farming in a sustainable food system. Proposed solutions range from ‘clean‐cow’ sustainable intensification to ‘no‐cow’, animal free futures, both of which encourage a disruptive break with past practice. This paper reviews the alternative proposition of regenerative agriculture that naturalises beef and dairy production by invoking the past to justify future, nature‐based solutions. Drawing on fieldwork in the UK, it first introduces two of the most prominent strands to this green rebranding of cattle: the naturalisation of ruminant methane emissions and the optimisation of soil carbon sequestration via the use of ruminant grazing animals. Subsequent thematic analysis outlines the three political strategies of post‐pastoral storytelling, political ecological baselining and a probiotic model of bovine biopolitics that perform this naturalisation. The conclusion assesses the potential and the risks of this approach to grounding the geographies and the temporalities of agricultural transition in the Anthropocene: an epoch in which time is out of joint and natures are multiple and non‐analogue, such that they provide slippery and contested grounds for political solutions.

## Graphical abstract

Anxieties around the relationship between livestock agriculture and the environmental crisis are driving profound and contentious discussions about the place of beef and dairy farming in sustainable food systems. This paper analyses efforts being made to reposition livestock animals as environmental allies. Under the regenerative agricultural banner, they are framed soil ecosystem engineers whose greenhouse gas emissions are natural and unproblematic.

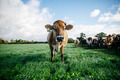

## 1 IT'S NOT THE COW, IT'S THE HOW

Growing concerns about the causal relationships between livestock agriculture and the environmental crisis are driving profound and contentious discussions about the place of beef and dairy farming in many parts of the world. High profile scientific papers and reports from multinational agencies have amplified existing concerns about the greenhouse gas (GHG) emissions from cattle. They draw attention to the links between livestock agriculture and land use change, deforestation and biodiversity loss. They flag water and air pollution and the risks of zoonotic disease and drug resistance (Poore & Nemecek, 2018; Willett et al., 2019). Some argue that *Livestock's long shadow* (Steinfeld et al., 2006) has grown to the point that we are living through *Apocalypse cow* (Gauvain, 2020).

Geographers have mapped several prominent ‘propositions’ (McGregor & Houston, 2018) emerging in response to this diagnosis. The first, commonly termed ‘sustainable intensification’, encourages accelerated, ecological modernisation to reduce the ‘ecological hoofprint’ (Weis, 2013) of cattle through modifications to breed, diets, pharmaceuticals and microbiomes (Godfray & Garnett, 2014). We are offered lean, efficient, ‘clean cattle’ optimised for environmentally friendly farming. A second proposition is found in growing calls to ‘de‐animalise’ the food system (Morris et al., 2019) by dramatically reducing the global population of cattle and shifting to ‘plant‐based’ or ‘lab grown’ meat and dairy alternatives. While arguments for plant‐based futures are long‐standing and come in diverse forms (Giraud, 2021), prominent advocates offer a new Big Veganism (Sexton et al., 2022) involving minimal consumer inconvenience due to the growing sophistication of meat and dairy substitution (Clay et al., 2020): burgers will still bleed and milk will still froth, but without the guilt. *Wow! No cow!* is the marketing slogan of one leading plant milk brand.

While the futures promised by these propositions differ markedly from the perspective of cattle, they have common advocates, like the US Breakthrough Institute, and share a temporality of transition that is premised on technological disruption; a radical break from an unsustainable past and a fast passage towards a hi‐tech, bright green and healthy future. Both the ‘no‐cow’ and the ‘clean‐cow’ futures celebrate the power of modern science, governance and capital to deliver increased production, fewer emissions and more space spared for wildlife (Guthman & Biltekoff, 2021).

In this paper we focus on a third proposition for solving the problems of livestock sustainability, identified by McGregor and Houston (2018) as the ‘naturalisation’ trajectory. Here, different representatives of the meat and dairy industries historicise, differentiate and naturalise modes of raising cattle to reposition their animals as traditional, environmentally friendly allies in the fight against climate change and biodiversity loss. In the words of advocates: *It's not the cow, it's the how*! (Rodgers & Wolf, 2020). Cattle are not wasteful ‘hooved locusts’,^1^ but ancestral and long abundant ‘keystone species’: surrogates for, or descendants of, the large herbivores that once roamed and shaped temperate landscapes. We trace how this proposition departs from the disruptive future orientation of the two previous propositions, by crafting a ‘future‐past’ (DeSilvey & Bartolini, 2019) – a retrospective invocation of an idealised former state to naturalise a simulated future – and explore how the grazing of cattle is legitimated through a careful reinvention of powerful pastoral imaginaries and baselines that promise to reconcile the interests of environmentalism and food production. New ‘carbon cowboys’ (Rodgers & Wolf, 2020) are emerging who choreograph holistic grazing systems to sequester carbon in the soil and to produce biodiversity. They promise ‘nature‐based solutions’, guided by new forms of ‘regenerative’ agricultural knowledge and practice.

In our analysis, we identify three political strategies through which this green rebranding of cattle occurs, which we term post‐pastoral storytelling, political ecological baselining and a probiotic mode of biopolitics. We define and explain these concepts, and offer them as a contribution to existing work in geography on the pasts and futures of livestock, that spans concerns with the place of cattle in both agriculture and conservation (Holloway et al., 2009; Lorimer & Driessen, 2016; Ormond, 2020). We are especially interested in the work done by nature‐based retrospection to legitimate a future for regenerative agriculture. Geographers and others have long been sceptical of grounding politics in a singular model of nature or natural science (Whatmore, 2002). And yet they have also noted that nature dies hard; it maintains popular valence and continues to provide discursive support to environmental projects in myriad shades of green (Castree, 2012). Debates about the futures of cattle offer a compelling microcosm of the current molten moment in the politics of nature in the Anthropocene: an epoch in which the modern great acceleration has put time out of joint, so that the familiar baselines that guide judgements of quality and authenticity have become unstable, and different actors reach back and forward in time to legitimate present actions (Latour, 2018). We offer these reflections as a contribution to an emerging critical dialogue in geography and elsewhere on the political and ecological potential of ‘nature‐based solutions’ as a new paradigm of environmental governance (Seddon et al., 2021).

This research forms part of a larger and longer running research programme on the social dimensions of livestock agriculture and its alternatives (Cusworth et al., 2021a, 2021b; Garnett et al., 2017; Godfray et al., 2018; Sexton et al., 2022). We ground the argument presented here in an analysis of stakeholder interviews with 28 individuals from a range of commercial, NGO, civil society, policy and scientific organisations, primarily located in the UK. These included those with explicit pro‐ or anti‐livestock agendas, as well as groups involved in food sustainability but with less partisan attitudes towards animal agriculture and meat and dairy consumption. We also spoke with those who work on the science of emissions calculations, and those involved in the development of relevant agri‐environmental policy. These interviews informed a textual analysis of select industry events, marketing materials, activism and journalism. This allowed us to locate where and why the claims made during the interviews had found traction in more public food and farming conversations. From these materials we were able to identify the key elements of a heterogeneous, yet internally coherent, retrospective, pro‐environment and pro‐ruminant food story. Discussions with interviewees centred on the UK agricultural sector and farmed environment. However, as we show, the new politics emerging around livestock sustainability involves significant traffic of ideas between the UK, North America, Australia, New Zealand and other EU Member States. The discussion we present here is thus intended to have application to those other geographies, not least because of the way the activities and historical referents relevant to one location are used to legitimate interventions in others. Finally, many of the issues and debates we detail here apply to other ruminants,^2^ including sheep, but we have decided to focus on cattle to streamline our argument.

## 2 THE GREEN REBRANDING OF CATTLE

The emerging proposition that supports regenerative agriculture entangles a broad range of arguments that span issues relating to food production and food consumption and includes concerns about both human and environmental health. Here we focus on what we consider to be the two most prominent strands to the green rebranding of cattle. The first is the naturalisation of ruminant methane emissions. The second is the optimisation of soil carbon sequestration via the use of ruminant grazing animals. We provide a short summary of each before moving to a thematic analysis.

### 2.1 Ruminant greenhouse gas emissions

**FIGURE 1:**
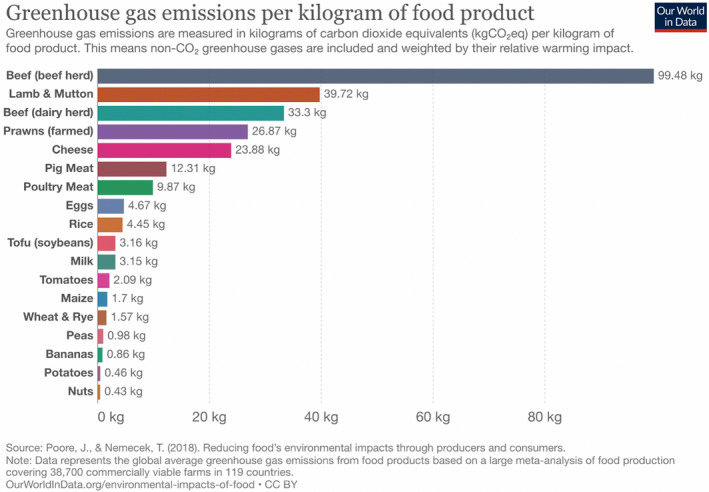
An our world in data graphic based on data from Poore and Nemecek (2018)

Relative to other foodstuffs, ruminant systems have a substantial and growing greenhouse gas emissions profile, including methane, carbon dioxide and nitrous oxide (Poore & Nemecek, 2018) (Figure 1). Methane is produced when ruminant animals digest their food, a process termed *enteric methane production*. Carbon dioxide emissions come from the production, processing and distribution of crops in systems that use compound feeds (i.e., where the animals are not fed exclusively on local grass, hay and silage). The intensive production of feed products – particularly soya – increases nitrous oxide emissions and is also a major driver of land use change and the release of stored carbon, particularly in forests in South America and South East Asia. Consequently, recent high‐profile reports, like that produced by the 2019 EAT‐Lancet Commission, which modelled globally sustainable diets, recommend a dramatic reduction in ruminant systems and the replacement of red meat with plant‐based proteins from legumes and nuts (Willett et al., 2019).

However, there is a growing sentiment within the livestock sector that the standard scientific tools – like those used in Figure 1 to commensurate the warming impacts of different GHGs – have overexaggerated the contribution that methane (and thus ruminant animals) makes to anthropogenic warming. These concerns have been fostered and given legitimacy by the publication of a new global warming potential (GWP) metric, entitled Global Warming Potential* (or GWP*). Those involved in the creation of this metric are concerned about the way GWP100, the de facto metric used in climate governance and emissions inventories, fails to capture the temporalities of warming of different GHGs, and thus misrepresents and exaggerates the effects of short‐lived climate pollutants like methane which get broken down in the atmosphere over relatively small periods of time (10–12 years) in comparison to carbon dioxide (100 s–1000s years) (Allen et al., 2018).

**FIGURE 2:**
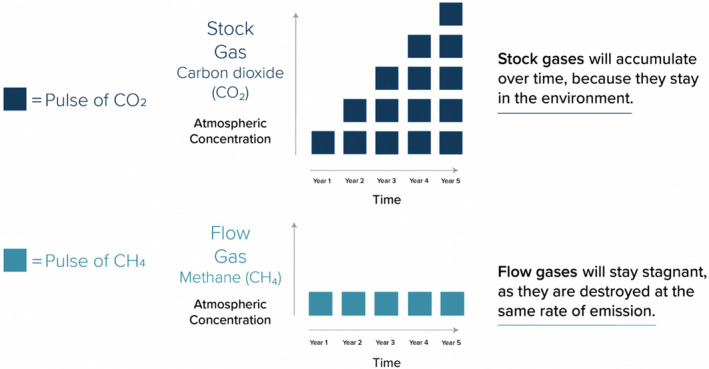
Stock and flow gases, taken from Mitloehner et al. (2020)

The creators of the new GWP* metric argue that over time periods relevant to the governance of anthropogenic warming, stable emission rates of short‐lived gases make no additional contribution to anthropogenic warming. This is because, when rates are stable, the atmospheric stock of the gas is replenished (via new emissions) at the same rate as it depleted (via atmospheric removal). So, if an emission of methane is part of a stable *rate* (e.g., from a herd of ruminants that stays the same size) then it does not cause any additional warming, it only maintains the warming caused by whatever activity caused the emissions rate in the first place (e.g., the building up of a herd of cattle). Methane is thus presented as ‘flow gas’ (Figure 2). These claims cannot be made for long‐lived ‘stock gases’ like carbon dioxide because over relevant time periods, emissions continue to accumulate in the atmosphere. Even when emission rates are stable, in other words, their warming impacts are cumulative.

It is argued that GWP100 cannot account for this temporal dynamic, because it evaluates the warming impacts of all different gases (including short‐lived gases like methane) in a way that isolates them from historical emissions. As a result, it is incapable of identifying when an emission of methane is part of a stable emission rate and thus causes no additional warming (Allen et al., 2018). GWP* navigates this problem by adopting a calculative system in which *pulses* of long‐lived climate pollutants are compared with *changes in the emission rates* of short‐lived climate pollutants. This allows the metric to identify when an emission of a short‐lived gas like methane is toggled to an atmospheric stock that is growing, shrinking or remaining the same, and thus whether the emission is having an additional, cooling or negligible impact on the climate.

The livestock industry has given substantial attention to arguing that cattle and sheep have been unfairly demonised by the GWP100 metric, and to suggesting that the GWP* metric can redress the balance (Mason, 2020; Smith, 2021), to the extent that some groups have lobbied the IPCC to adopt GWP* as a new metric for GHG accounting (NFU, 2020b). In their discussion of this debate, the IPCC recognises that metric design has a non‐trivial influence on the distribution of warming responsibility and conclude that decisions about selecting the metrics used to underpin climate governance need to be taken by political rather than technical organisations (IPCC, 2021). GWP100 remains the de facto metric in emissions inventories and trading schemes.

One central plank in this argument is the claim that the methane produced by contemporary livestock animals is a continuation of the methane produced by large herds of ruminant grazers in the past. Under this calculus, the now diminished populations of animals like bison, buffalo and aurochs, that roamed temperature regions before the agricultural revolution, create an allowance of methane that modern agricultural animals are simply fulfilling (e.g., Kelliher & Clark, 2010). Others point to the substantial historic presence of livestock throughout agriculture's pre‐industrial and industrialising past (Liu et al., 2021). Although this baselining activity was underway before the advent of GWP*, the metric has powered up the arguments of those seeking to naturalise and legitimate the emissions produced by livestock *now* via reference to the emissions produced by animals *then*. Advocates suggest that so long as ruminant methane emissions are kept constant then cattle cause no additional warming.

Furthermore, GWP* shows how decreasing rates of methane emissions, when set against historic baselines, can confer a cooling effect because the atmospheric stock is depleted quicker than it is replenished. This revised calculative framework can cast ongoing efforts to improve the methane efficiency of livestock farming, which reduce the amount of methane emitted per unit of food produced, in a new and even more favourable light (Ridoutt, 2021b). Advocates suggest that by achieving these incremental emission reductions, these technologies can help livestock systems become a warming‐neutral, greenhouse gas mitigation and sequestration solution (Mitloehner et al., 2020).

These claims have had traction amongst advocates of extensive ‘pasture‐based’ systems who are keen to differentiate their operations from more intensive beef and dairy production systems. We are told how the emissions on pasture‐fed systems come almost exclusively from the animals' enteric methane production and so the warming attributed to them is substantially reduced when using GWP* (see, for example, NFU, 2020a). In contrast, the emissions breakdown of more intensive systems that use more compound feed products includes a greater portion of long‐lived emissions like nitrous oxide and carbon dioxide, due to fertiliser use, transportation and land use change associated with the production of feed crops. As a result, intensive operations continue to have a substantial warming footprint even under alternative warming metrics (Lynch, 2019).

### 2.2 Soil carbon

The second strand of the rebranding of ruminant animals reimagines their role in the management of soil health and the delivery of biodiversity. Soil degradation and erosion are well‐established concerns for those flagging the unsustainability of the modern food system (Doran, 2002). This degradation is linked to excessive tillage, pesticide and artificial fertiliser application, the simplification of crop rotations, and the separation of livestock and arable farming (Pimentel & Kounang, 1998). These practices, which are central to intensive agricultural management, disturb soil structure, expose it to erosion, compromise its complex ecosystem, and reduce its fertility, organic matter content and ability to retain moisture (Lal, 2015). Although such claims are contested, and there is great variation across soil types and farm systems, campaigners frequently caution that agricultural soils only have a few harvests left (Ritchie, 2021).

**FIGURE 3:**
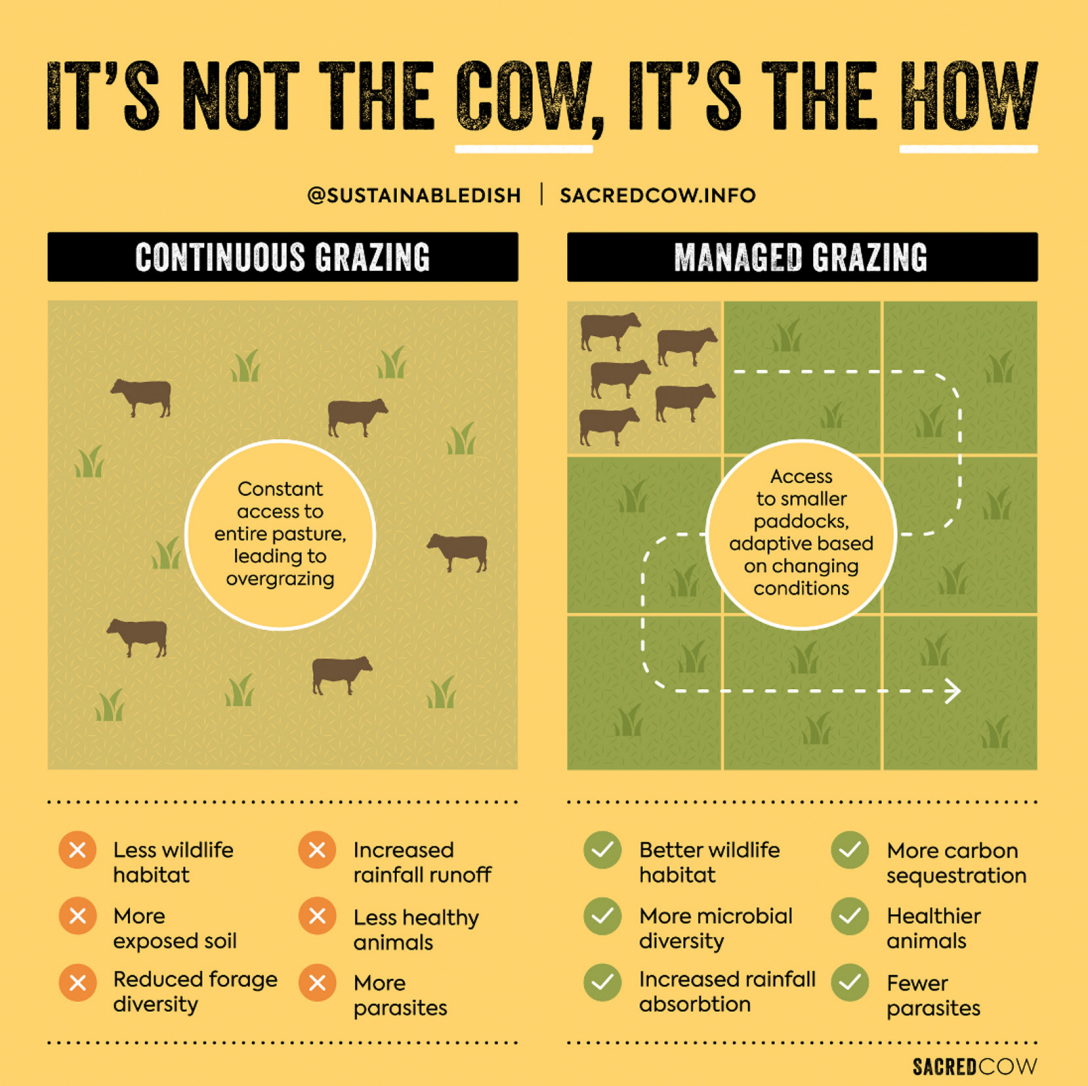
The principles and advantages of managed grazing systems, taken from the sacred cow website, associated with the book and the documentary film of the same name (Rodgers & Wolf, 2020)

In response, advocates for ruminant grazers have begun reframing their livestock as vital actors for the regeneration and sustainable management of the farmed environment. They build on claims of the ancestral provenance of pasture‐based livestock systems to flag the ecological role of cattle in creating grazed landscapes rich in biodiversity and the role of grazing in generating soil health. To maximise these environmental benefits and to optimise agricultural productivity, regenerative farmers have developed a set of ‘holistic’, ‘mob’, or ‘rotational’ grazing practices. These typically involve choreographing the movement and grazing of animals around small ‘cells’ or parcels on the farm (Figure 3). The aim is to keep the grass at the stage in its growth cycle with the highest rates of photosynthesis and root growth to maximise soil carbon sequestration, to gently disturb the grass and trample the manure into the ground to provide habitat diversity for different worms and insects, and to improve soil mineral content (Flack & Karreman, 2016).

In mixed livestock‐arable systems, ruminant grazers are particularly valued for their ability to fertilise the soil with their manure, which helps increase feed insects and microbes, to enhance the productivity of successive crops and/or reduce dependence on financially and environmentally costly synthetic fertilisers. The presence of cattle on the farm also facilitates the inclusion of temporary grass, cover crops and herbal lays in the agricultural rotation. These practices are employed to reduce soil disturbance via ploughing, to create a more diverse portfolio of agricultural land use, and to retain plant cover on the ground throughout the agricultural calendar. All these interventions aim to minimise erosion and to increase soil depth, structure organic content and thus fertility.

Although such practices were once common in agricultural management across the UK, Europe and North America, the intensification of agriculture, particularly since the Second World War, has seen the specialisation and separation of arable and livestock production and the simplification of crop rotations (Bowler, 1986). The (re) integration of livestock animals into arable systems is one of the major motifs of the regenerative agricultural movement. But these practices of reintegration are indexed to an older pre‐historical baseline. We are told that they simulate the browsing, eating and digestive behaviours of the ancestral herds of free roaming ruminants, now confined to smaller areas of land (Briske et al., 2011).

Many of these claims are long‐standing, but in the last decade this green rebranding has acquired a new climate‐related dimension, in which ruminant animals are promoted for their ability to maximise the amount of carbon that is sequestered into the soil. Under this rationale, soils subject to naturalistic grazing become the locus for a ‘nature‐based solution’ for offsetting carbon dioxide emissions. By sinking carbon in soils through the regeneration of degraded land, cattle (it is argued) can contribute to strategies towards net zero, or even negative emissions. While some evidence is emerging about the carbon sequestration potential of certain grazing systems at least in the short term (Rowntree et al., 2020), questions remain about the extent to which this carbon can be sequestered continuously and indefinitely, the extent to which any benefits arising from sequestration achieved are offset by the fact that these grazing systems are very land demanding, and the degree to which animals are actually necessary to secure high levels of sequestration (Briske et al., 2011). Current evidence suggests that grasslands grazed by livestock have an overall net warming effect unlike those that are unmanaged or sparsely grazed (Chang et al., 2021).

In linking soil health and biodiversity provisioning to traditional pastoral and mixed farming systems, some in the livestock sector have sought to challenge the claims made about the environmental credentials of no‐cow new veganism. They suggest that the intensive ‘plantation’ systems that commonly underpin the production of crops like wheat, soya and corn that go into ‘ultra‐processed’ vegan foods have major impacts on soil health, are heavily dependent on synthetic fertilisers and threaten local livelihoods (Sexton et al., 2022). They argue that it is disingenuous to say that a vegan diet is a satisfying solution to the social and environmental crises in the food system (Maye et al., 2021). Pasture‐fed livestock systems, by contrast, are positioned as more natural (in that they displace the need for synthetic fertilisers and foster good soil health) and traditional (in that they resemble the once‐common livestock‐arable mixed management or extensive grazing systems) whose food products do not rely on intensive agricultural commodities produced through obscure and unjust supply chains (Rebanks, 2020).

## 3 NATURE AT WORK

It is clear, even from this brief synopsis, how powerful, retrospective ideas of nature and the natural are being put to work by these carbon cowboys to both contest emerging clean cow and plant‐based futures and to naturalise their regenerative alternatives. In the analysis that follows, we identify three strategies that we consider to be central to this politics of nature.

### 3.1 Post‐pastoral storytelling

Idealised imaginations of the rural pastoral have long served some urban citizens in Europe, North America and elsewhere as the nostalgic counterpoint to the perceived social, ecological and economic depredations of modern life (Marx, 2000; Williams, 1973). Geographers and others have observed how premodern landscapes, replete with contented livestock, cowboys, stockmen and shepherds serve as idealised normative benchmarks for aesthetic, cultural and recreational experience (Woods, 2010), featuring prominently in literature and film (McHugh, 2011). Those involved in the production and marketing of meat and dairy have long made recourse to this pastoral discourse, mobilising the traditionality of livestock management systems to justify their role in shaping the countryside and invoking the ‘naturalness’, ‘terroir’ or ‘localness’ of meat and dairy to shore up their place in the national diet (Dupuis, 2002; Fiddes, 2004).

However, the green rebranding of cattle shows a novel mutation in this discourse; a compelling illustration of what the anthropologist Heather Paxson (2012) terms the ‘post‐pastoral’; a discourse that has come to characterise agriculture and food narratives in powerful parts of the post‐industrial Western world. Paxson develops the concept of the post‐pastoral from the literary critic Terry Gifford (2006), to describe the subtle reworkings of the pastoral narrative by producers and consumers who are coming to terms with the environmental and social crises of modern agriculture, in her case artisan cheese makers in gentrified parts of rural New England. She traces how adherents of the post‐pastoral tone down the anti‐modern, anti‐urban tenor of the pastoral ideal, maintaining the importance of a working landscape, while reinventing pastoral practices, knowledges and forms of economic activity. In particular, post‐pastoralists seek to retain the cultural allure of craft and a proximity to nature, while embracing the power of science and technology, and reasserting the place of capitalism.

In the case of the green rebranding of cattle, crafting the post‐pastoral is firstly an ontological project. Cattle cowboys operate in a liminal space ‘after nature’ in the sense described by Marilyn Strathern (1992). As Paxson explains they are ‘at once post‐nature, recognizing that there is no pristine natural world outside human cultural activity, and also ever in pursuit of some kind of remade nature as a ground for appropriate human action’ (2012: 18). Cattle cowboys depart from the binary idea of a pure nature that could be separated from society and ‘spared’ (Green et al., 2005) through a disruptive transition to either a lean cow or a no‐cow future. Instead, according to one industry publication, cattle and people are ‘a part of nature's carbon cycle’ (Bauer, 2018) and can form mutualistic relationships for land sharing. As we discuss in more detail below, the cowboy and domestic cattle are folded into this naturalistic ontology as ancestral, landscape‐shaping ‘keystone species’. This systemic, circular and non‐binary ontology is central to the explanatory frameworks of climate and soil science that underpin the methane and soil carbon story.

**FIGURE 4:**
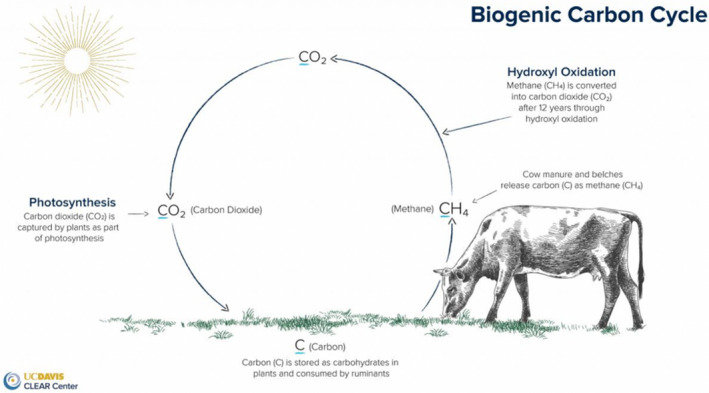
The biogenic carbon cycle, taken from Mitloehner et al. (2020)

We find one example of this post‐pastoral ontology in the conceptualisation of cattle emissions and their role in soil carbon sequestration as ‘biogenic’. The term, which has become popular amongst carbon cowboys and those otherwise invested in the regenerative potential of ruminant animals, is used to differentiate between the release of carbon from stable reserves in the form of fossil fuels, and the cycling of gases and energy actuated through processes of respiration, digestion and photosynthesis (Figure 4). Via the process of atmospheric removal, methane is broken down after 10–12 years back into the same quantity of carbon dioxide that was in the atmosphere before being photosynthesised by the grass, and before being digested by the ruminant animals.

In this biogenic framework, the warming impact of the methane produced by ruminants is a natural feature of a system in benign stability, while the warming associated with activities reliant on fossil fuels are not, as they add new carbon into the system that would otherwise have been locked away in inert forms like coal, oil and gas. This distinction is frequently reiterated by those in the livestock sector who claim that not enough attention has been paid to the warming impact of fossil fuels, and that excessive scrutiny has been placed on the warming impacts of meat and dairy production (e.g. Mitloehner, 2020). Biogenic methane is, in this reading, natural, inevitable and unproblematic; and the common tools like GWP100 that are used to inform climate governance have helped create ‘bogus burger blame’ (Mitloehner, 2021).

Although it folds domestic cattle into a naturalistic ontology, regenerative agriculture also permits an important role for modern technology. While carbon cowboys might bemoan the monstrous character of some industrial plant‐based proteins and summon fear of the ‘fake meat’ produced through cellular agriculture (e.g., Niman, 2020), they do not advocate a Luddite retreat to traditional technological regimes. Instead, some post‐pastoral mob grazers deploy cutting‐edge digital technologies to choreograph the naturalistic movements of their cattle. They use GIS software to divide up their land into cells and combine climate, soil and vegetation data sourced remotely from drones and satellites with that gathered by their tractor‐mounted computer systems to strategically plan which areas will be used in rotational grazing. The subsequent locations and movements of cattle are controlled either through mobile electric fencing or by ‘NoFence’ virtual fencing using GPS‐located electric pulse collars. Here regenerative practitioners are both modernising an ecologically sensitive agricultural epistemology, whilst ecologising modern agricultural technology (Kearnes & Rickards, 2020). In doing so, they are shifting the position of their tools and gadgets in relation to the pastoral ideal from the degenerative ‘machine in the garden’ (Marx, 2000) towards a post‐pastoral figure of the ‘machine as gardener’ (Cantrell et al., 2017); repurposing the very technologies that have enabled agricultural intensification as part of a holistic, remedial programme for regeneration.

Epistemologically, the green rebranding of cattle is characterised by a post‐pastoral blending of scientific and folk knowledge, seeking to harness the cultural allure of the pastoral narrative to the political and economic power of scientific metrics and calculation. A popular example is offered by a social media advert for the fast‐food company *Burger King*, who have recently recognised the marketing potential of the idea of regenerative agriculture. This short film is set in cartoon ‘low‐carbon land,’ in which the 11‐year‐old country music star Mason Ramsey sings:

When cows fart and burp and splatter,
well it ain't no laughing matter—.
they are releasing methane every time they do.
And that methane from their rear.
goes up to the atmosphere.
and pollutes our planet, warming me and you!
Yes! That methane that they pass.
is a greenhouse gas.
that'll trap the sun's heat n' change our climate, too!
To change their emissions,
Burger King went on a mission.
testing diets that would help reduce their farts,
That's a start!
And by now there ain't no question.
that it's helping cows' digestion.
adding lemongrass, so they can play their part.
Reducing methaaaaaane… methaaaaaane.
And the scientists have proven that it works …

The lyrics follow a catchy Country and Western tune and blend comic scatology with technical and scientific detail about methods of methane reduction. The iconography moves from a cartoon feedlot with farting cattle, through a dystopian weather event, to children in white lab coats dancing on melting ice and holding lemongrass plants. It finishes in a post‐pastoral rural idyll; a fairground full of happy healthy children having fun in cowboy costumes. In the post‐pastoral logic of the advert, we are to be reassured that the folksy rural values associated with this burger brand are now buttressed by science, and amenable to technological enhancement. The advert aims to build trust in a global beef brand; tapping into, and further accelerating, the globalisation of the iconography of the American cowboy, while crafting a generic model of the regenerative cow and a standardised model of regenerative farming practice (for further discussion, see Turnbull & Oliver, 2020). We discuss these trends in more detail below, which offer a striking example of what Morris and Reed (2007) have described as the ‘McDonaldization of on farm conservation’.

### 3.2 Political ecological baselining

Perhaps the most compelling illustration of this post‐pastoral reconciliation of folk wisdom with natural science is found in the retrospective futurology that is central to the green rebranding of cattle and the derivation of desired future‐pasts. This involves a process we describe as ‘political ecological baselining’: invoking a valued past to naturalise present projects that aim to deliver desired futures. The concept of the ecological baseline has risen to prominence in ecology and natural resource management and is the subject of a growing body of critical work in the social sciences (Ureta et al., 2020). One origin story ties the concept to a growing awareness of what the fisheries scientist Daniel Pauly (1995) described as the ‘shifting baseline syndrome’. Here contemporary resource managers fail to identify the past effects of human activities when setting their ideal for what they consider to be a natural ecology. Consequently, the degraded present is normalised, and past ecological patterns, processes and abundance are neglected. Naming this condition as a ‘syndrome’ is key to its political framing within a retrospective narrative temporality, in which a wise ‘seer’ draws on their knowledge of the (often deep) past to diagnose a problem in the present, and to advocate future solutions that require a return to, or a reinvigoration of, past practices. This technique of back and future casting involves the identification of ‘usable pasts’ (DeSilvey & Bartolini, 2019) that serve as ‘anticipatory baselines’ (Hirsch, 2020) to legitimate and help enact desired futures.

We can see manifestations of this political ecological baselining in our two examples of green rebranding. In the first, efforts to naturalise contemporary ruminant methane emissions benchmark the present to a historical baseline characterised by large herds of large herbivores grazing and burping at a scale comparable with contemporary cattle populations. The historical date of this baseline is generally poorly specified but harks variably back to periods before the ‘Pleistocene overkill’ (Martin & Klein, 1984) (c.12–15,000 BP), widespread cattle domestication (10,000 BP), and the ravages of hunting that accompanied the agricultural revolution and colonial expansion (150–500 BP).

**FIGURE 5:**
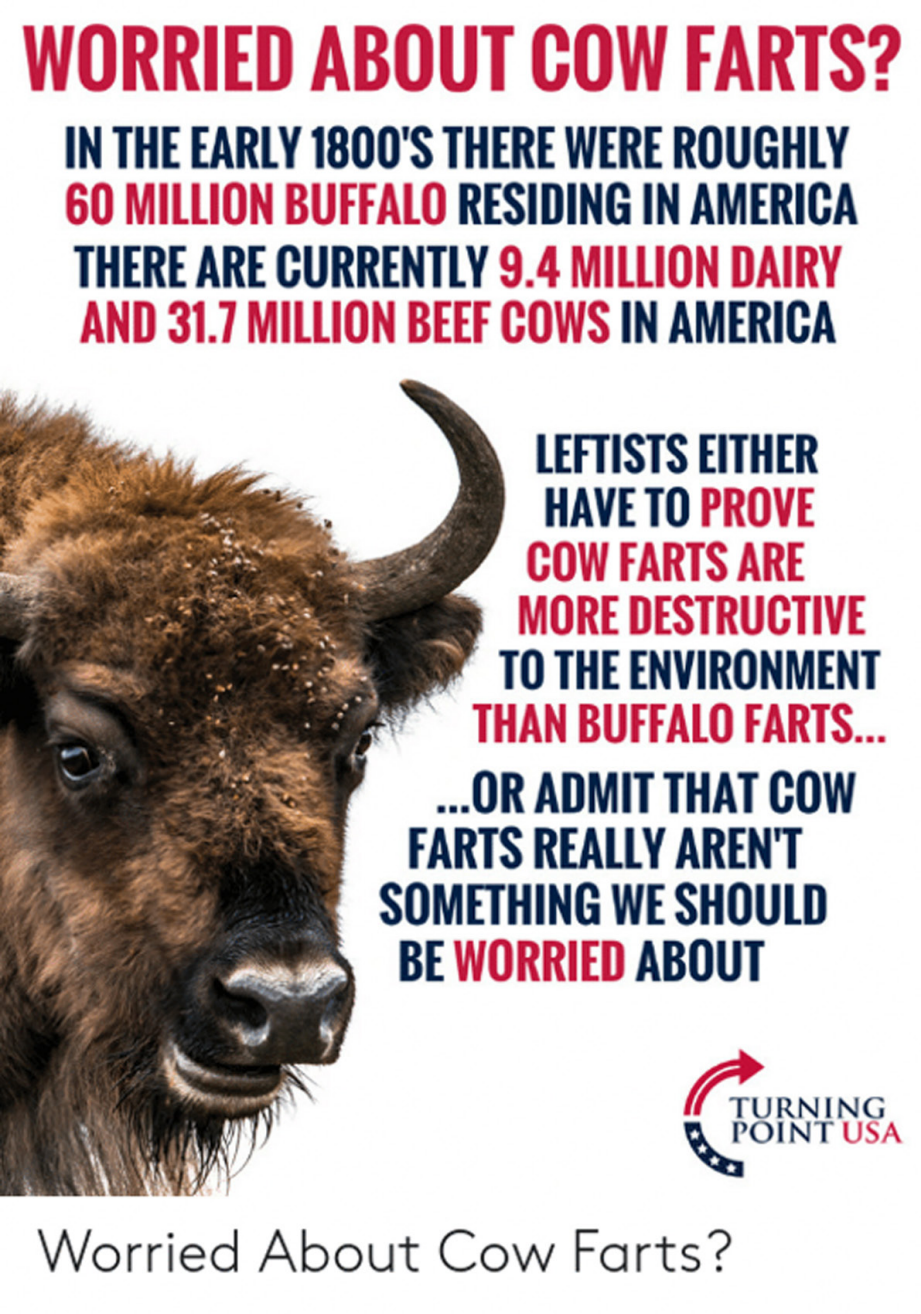
Image taken from Turning Point USA (2019)

Either way, we are reassured that there were once far more wild herbivores, and this baseline helps downplay the environmental significance of contemporary emissions. For example, the AgResearch group that ‘partners with the pastoral sector’, compares the methane emissions of historical bison herds with agricultural cattle in the United States. They suggest that the 30 million bison estimated to have roamed the Great Plains produced 2.2 tg of methane per year, whilst the 36.5 million cattle located in the ten states covering the historic bison range produced 2.5 tg of methane per year (Kelliher & Clark, 2010). This argument is captured and politicised in Figure 5, an image produced and posted in 2019 by the right‐wing advocacy organisation Turning Point USA (2019) (see also Brown, 2018; Savory & Butterfield, 2016).

Here GWP* provides the metrological framework for backcasting to a politically salient environmental baseline to ground a positive evaluation of the warming impacts of contemporary emissions. We are told that the long‐standing presence of ruminants in the food system downgrades their perceived warming culpability, so long as ruminant numbers fall or remain constant as they have done for some time in the UK, in North America and in the EU.

A political ecological baseline characterised by abundant herbivores also features prominently in the second example of soil carbon sequestration. The baseline in this story is a premodern ecology, in which free‐ranging herbivores, left to their own devices, unfettered by fencing, and grazing in the ‘ecology of fear’ provided by predators like wolves, would have moved widely across landscapes, grazing intensively, but selectively and sporadically, and defecating copiously in ways that would have generated biodiversity and led to the accumulation of deep, nutrient rich, structurally complex and resilient soils (Hillenbrand et al., 2019). Contemporary advocates suggest that hardy breeds of cattle can serve as contemporary ecological surrogates for these wild ancestors. Their ‘naturalistic’ grazing regimes mimic some dimensions of these historical practices and thus enhance biodiversity and carbon sequestration, while still producing good quality ‘pasture‐fed’ beef and dairy.

These principles have had particular traction in the United States and sub‐Saharan Africa, where high‐profile farm celebrities including Gabe Brown, Joel Salatin and Allan Savoury, promote regenerative grazing for revitalising grassy plains to their pre‐colonial condition (often without engaging with questions of political and racial justice; Philpott, 2020). Such individuals rely heavily on visual representations of their management practices, including high‐profile documentaries like Netflix's *Kiss the ground* (Tickell & Tickell, 2020), and social media to network with other practitioners. These ideas have taken root in parts of Europe, where advocates of a retrospective model of regenerative beef and dairy production find allies in some parts of the burgeoning rewilding movement, who have successfully made a case for the back‐breeding, de‐domestication and reintroduction of large herbivores as key agents in the restoration of functional ecosystems. Hybrid models are emerging of agricultural wilding – for example at Knepp in the UK – in which hardy breeds of ‘native’ cattle are given substantial autonomy while still being slaughtered for beef (Lorimer, 2020). The political ecological baselining of bovine methane emissions and soil carbon sequestration are often folded together so that the aurochs and bison of prehistoric ecologies create a methane allowance for contemporary livestock and provide guidelines for the optimisation of soil carbon sequestration.

This baselining has two political consequences that are important for our analysis. It first works to establish and quantify a natural, historical ecological hoofprint to be used to inform the audit of contemporary practices and the setting of targets for future emissions reductions. In so doing, it facilitates the construction of a more generous warming profile for livestock, and then flags how livestock can play a part in achieving net zero, or even in delivering negative emissions. It is argued that cattle can become part of the solution by reducing methane emission rates (through dietary/microbial interventions or capture and storage) and/or by enabling soil carbon sequestration. In research funded by Meat and Dairy Australia, for example, metrics better equipped to integrate historical emissions (namely GWP* and Radiative Forcing analyses) are used in conjunction with evaluations of the carbon sequestration of grazing land to demonstrate the warming‐neutral (Ridoutt, 2021a) or even cooling (Ridoutt, 2021b) impact of livestock systems.

Second, this act of baselining has been used to establish a generic historical nature whose value and authenticity is indexed less to specific places and more to its conformity with GHG metrics and regenerative practices. As a standardised ‘global nature’ (Franklin et al., 2000), regenerative agriculture has proved amenable to globalisation, becoming mobile along the international networks that enable agricultural governance to further the McDonaldisation of on farm conservation. Perhaps the most compelling example of this geography is offered by the globalisation of mob grazing. The practice, which started with the work of Alan Savoury to emulate the ecologies (though not the political economy) of pre‐colonial sub‐Saharan Africa, now has advocates in the United States and the United Kingdom who have translocated imaginaries, practices and even desired organisms into locations marked by a great deal of climatic, ecological and political variation.

There is a common geographic flexibility granted to the origins of the species that are used as surrogates for wild herbivores in contemporary regenerative systems. While the ruminant‐as‐ecological‐surrogate model takes us back to the early years of the Holocene, it is very much designed to anticipate the non‐analogue geographies and temporalities of the Anthropocene (cf. Ureta et al., 2020). In the North American context, cattle are naturalised via reference to their bison ruminant relations, and their analogous impacts on the landscape. But there are non‐trivial physiological and ecological differences that can be overlooked in these substitutions. For example, bison co‐evolved with the soils and vegetation of the prairies, roam large areas and have hooves that tread lightly on the grass, while cattle, which are much later colonial imports, tend to stay around smaller and wetter areas and have hooves that can damage the landscape (Steuter & Hidinger, 1999).

Although there is variation in the sorts of regenerative practices adopted in different locations and advocates stress the need for individuals to experiment with regenerative practices to see which work on their farm and which do not (Giller et al., 2021), there is nevertheless a general convergence on a small number of practices. This McDonaldisation of regenerative grazing reaches its apogee in Australia. Despite it being a country with only a very recent colonial history of ruminant livestock agriculture, and thus a place where it might be argued that cattle ‘do not belong’ (Saltzman et al., 2011), it boasts some of the loudest voices championing the natural role of ruminant animals in the safeguarding of the farmed environment. Here the green rebranding of cattle involves either subsuming the national cattle herd into a dislocated past global herbivore population or proving that the ecological processes that link ruminants to good soil health pertain to Australian landscapes (see the Youtube channel ‘Australian Good Meat’, for example).

The convergence and divergence between the animals present in pre‐human ecologies and contemporary farmed landscapes has a long and troubled history. The exportation of pastoral agricultural practices naturalised the presence of settler populations, and justified their ongoing civilising missions (Gillespie & Narayanan, 2020). Although we are wary of making direct links between these colonial legacies and the regenerative movement, it is important to note the histories that are invoked when pastoral ideals and norms of good (livestock) farm practice are trafficked around the world (Sayre, 2018). Likewise, under the Green Revolution, modern, Western and capitalist knowledge practices from the Global North were regarded as more legitimate than Indigenous or vernacular farming knowledge in the Global South (Shiva, 1991). Although the character of the agronomic practices may be different in the regenerative movement to those championed for mid‐twentieth‐century agricultural productivism, there is a danger that a select cohort of experts will assume primacy over the knowledge‐claims of local farming communities.

### 3.3 A probiotic model of bovine biopolitics

Finally, the green rebranding of cattle emissions through this post‐pastoral storytelling and political ecological baselining enacts a novel mode of ‘bovine biopolitics’ (Lorimer & Driessen, 2013). This concept describes how the lives of cattle and livestock farmers, and the wider pastoral landscape, are known and governed through the application of knowledge about cattle and their care. Existing work in geography has mapped how cattle and farmers have been made subject to the contrasting biopolitical logics of intensive production, animal welfare, biosecurity and heritage breed conservation (Cole, 2011; Hinchliffe & Ward, 2014; Holloway et al., 2009). Here we see the emergence of a different model with distinct consequences for both the animals and the people it makes subject, which we discuss in turn.

Regenerative cattle figure as both ancestral ‘keystone species’ and as prospective ‘ecological engineers’ – two terms with slightly divergent meanings and biopolitical consequences. As keystone species, cattle are understood to be endowed with disproportionate ecological agency by virtue of their position in the food chain, and their ability to instigate ecological disturbance regimes. When grazing and defecating naturalistically as surrogates for the aurochs, cattle promise to catalyse ecological restoration, producing enhanced plant and microbial biodiversity and delivering ecosystem functions at a landscape scale. As ecological engineers, cattle are put to work on a larger, planetary scale. They are understood to be endowed with the ability to control the global atmospheric circulation of greenhouse gases, by modulating gaseous fluxes through carbon sequestration and methane emissions reductions. This framing dominates, by way of example, the Netflix *Kiss the ground* documentary. As keystones and engineers, cattle are paradigmatic of the small number of plant and animal species that are currently being charged with delivering nature‐based solutions. They figure alongside fungi (Oviatt, 2021), oysters (Wakefield, 2019), beavers (Lorimer, 2020) and assorted tree species (Palmer, 2021) as living tools for landscape and planetary‐scale geoengineering. They become charismatic icons and hardworking allies in global stewardship projects to repurpose agriculture to help achieve net zero targets.

Similar to the role of legumes in regenerative farm management (Cusworth et al., 2021a), cattle are enlisted as agents of a ‘probiotic’ (Lorimer, 2017) mode ‘environmental’ biopower (Braun, 2014). This aims to address the pathological consequences of modern antibiotic modes of managing life and the ‘Anthropocene blowback’ to which these have given rise (Guthman, 2019; Hinchliffe et al., 2016). This probiotic mode of bovine biopolitics is geared less towards securing the size, health and identity of animal breeds to enable protein production, and more towards the modulation of earth systems to deliver desired processes and functions. Herds of cattle become agents for the modulation of planetary metabolism, while at the same time finding their own internal metabolisms subject to further genetic and ecological modulation (Ormond, 2020). As trans‐scalar agents of this ‘metabo‐politics’ (Folkers & Opitz, 2022; Turnbull & Oliver, 2020), regenerative cattle come to sit somewhat uneasily between the logics of modern agriculture and of rewilding. While they are granted more autonomy than those kept in the feedlot, their individual and aggregate anatomies, behaviours and metabolisms are optimised for the provision of low‐carbon meat and dairy.

The pastoral tradition places great value on the moral virtue of working human and animal bodies. In keeping with this vision, individual cattle are subjectified as a novel type of probiotic ‘animal labourer’ (Barua, 2019) whose work conjoins the ‘metabolic labour’ (Beldo, 2017) associated with intensive meat and dairy production and the ‘ecological labour’ associated with keystone species charged with solving anthropogenic environmental problems (Lorimer, 2020). Advocates for regenerative agriculture naturalise the ideas of the working animal and the wider ‘work of nature’ (cf. Besky & Blanchette, 2019) through discursive reference to the long‐standing pastoral ideal of the ‘working landscape’ (Marx, 2000; Paxson, 2012). For example, the British celebrity shepherd James Rebanks (2020) informs us that regenerative farmers in the Lake District are ‘working with nature’, returning to the traditional methods of animal husbandry practised before the Second World War that produced the world‐famous cultural landscape. In some cases, this discourse of traditional work interfaces with claims for improved animal welfare, in which cattle are granted more autonomy and work in relations that better approximate historical and more beneficent modes of domestication (Porcher, 2017).

This novel mode of bovine biopolitics also has implications for the subjectivities of farmers and others in the livestock food system. The discourse of putting nature to work serves to fold the carbon cowboy into ecological systems as the choreographer of natural dynamics; a human ‘hyper‐keystone species’ in the terms of Robert Paine (the original inventor of the concept of the keystone species; see Worm & Paine, 2016). As an enlightened hyper‐keystone, the carbon cowboy sits at the very apex of the food chain. They are endowed with the power and responsibility of shaping the behaviours and abundance of all other species below them, and are thus ultimately able to secure the efficient operation of the ‘biogenic’ circle of life.

This naturalisation of the carbon cowboy serves to challenge emerging alternative subjectivities which also call upon the past to naturalise their normality. The first is a retrospective, yet future‐orientated vegan argument that draws on strands of anthropology to demonstrate that popular narratives about prehistoric meat eating are both greatly exaggerated and gendered since women's foraging activities provided most of the calories (Dunn, 2012). The second is common to the growing ‘paleo’ movement for whom modern Westerners are alienated from their ‘evolutionarily adapted environment’: a political ecological baseline full of brave noble savages fighting, killing and eating a violent nature that was displaced by the rise of livestock agriculture, and the sedentary lifestyles and processed food diets to which it gave rise (Leiper, 2019; Zuk, 2013). In contrast, the subject position of the carbon cowboy offers a conservative futurology that naturalises domestication, livestock and meat‐eating, and figures the cowboy as both the right type of (meat) eater as an enlightened planetary steward.

Finally, as McGregor et al. (2021) argue in their analysis of the biopolitics of cattle methane emissions reduction, this probiotic biopolitics also affirms an individualised ‘green governmentality’ centred on the consumer‐citizen, who must be engaged and empowered to make rational, ecological decisions, and economically secure enough to act on them. This is also the case with no‐cow Big Veganism (Sexton et al., 2022). While producers and retailers supporting regenerative agriculture may be cautiously willing to engage in the model of ‘less and better’ meat, they have done much more to encourage the latter than the former (Trewern et al., 2021). As the discussions around livestock at the latest UN Climate Change Conference (COP26) demonstrated, meat reduction and dietary change remain politically unpalatable. Although global methane emissions per cow have dropped over time due to changes to diets, breeds and microbiomes, total emissions have increased – and are projected to keep on doing so – as global demand rises (Reisinger et al., 2021).

## 4 CONCLUSIONS: WHERE'S THE BEEF?

In this paper we have reviewed an emerging proposition in current debates about the futures of livestock agriculture in the context of growing concerns about climate change and biodiversity loss. We traced the green rebranding of cattle in response to prominent appeals for both the sustainable intensification and the de‐animalisation of agriculture, focusing on contestation around cattle methane emissions and their potential role in soil carbon sequestration. We explored how invocations of *nature* and *the past* are called on to justify future interventions. While advocates for cellular agriculture and plant‐based futures herald the potential for a radical break from an unsustainable status quo, our cattle cowboys curate a retrospective narrative that finds value in a pastoral past, and which serves to legitimate the continuation of a reformed present. In our analysis we identified three central political strategies, which we termed post‐pastoral storytelling, political ecological baselining and a probiotic model of bovine biopolitics. In all three, appeals to past nature play a powerful political role in buttressing affirmative claims for cattle and in undermining appeals for plant‐based futures. In conclusion we briefly reflect on what is at stake in this debate over the nature and future of agriculture, and explore what risks being effaced through this politics of nature.

We start with a note of caution. Regenerative agriculture is a label endogenous to the agricultural sector, rather than one imposed and certified from the outside. This makes it interesting and important, but it is also extremely heterogeneous. The figure of regenerative cattle is capacious, enfolding interventions as diverse as probiotic feed supplements – as in the case of the Burger King advert – to the release of herds of hardy cattle as naturalistic wild grazers, for example at Knepp in the UK. At times regenerative agriculture overlaps with the clean cow model of sustainable intensification, in other manifestations it offers a radical departure. This diversity permits and potentially naturalises a great diversity of modes of husbandry and of political economic forms. We find that regenerative agriculture itself remains poorly defined (Giller et al., 2021), its future is underdetermined, and it merits further study. Nonetheless, our analysis suggests enough commonality in this green rebranding that we can identify shared promises and risks, as well as identify priorities for future research.

Geographers are familiar with the risk that appeals to nature and to the past simplify complex debates and narrow the heterogeneous mix of possible futures that will be necessary to enable just transitions in the food system (Guthman, 2014). The same is also true for demands – such as those made by advocates for a new veganism or sustainable intensification – for a clean break, disruptive transition to clean‐cow, animal‐free futures. These accelerationist acts of futuring risk effacing the important ecological, political and cultural legacies of livestock farming (cf. Goldstein, 2018). On one reading, the retrospective temporality favoured by carbon cowboys risks negating the political work required to deliberate as to its relative merits: nature is used to shortcut politics. But our analysis suggests that its adherence to a post‐pastoral naturalism does enable more promising political ecological temporalities, in which quality is not directly indexed to past authenticity or to the speed of change. In the reinvention of the pastoral offered here, there is a place for science, for technology, for markets and for other late modern political technologies. The future‐past of regenerative agriculture does offer a viable mode of baselining for the non‐analogue conditions of the Anthropocene, but it needs to engage more coherently with its political dimensions.

In particular, our analysis suggests that the emerging mainstream green brand of regenerative agriculture risks globalising a generic future‐past that is disembedded from: (i) histories of land acquisition and management; (ii) the place of food production; and (iii) the socio‐spatial relations that underpin agricultural supply chains. It performs a process we describe as the McDonaldisation of regenerative agriculture. In contrast, research on agro‐ecology – an older cousin of regenerative agriculture – points to myriad local models of food production North and South, some of which include livestock, that are tailored to local climatic and ecological conditions, as well as to local markets and modes of food sovereignty (Holt‐Giménez & Altieri, 2013). Agro‐ecology shares many of the production principles of regenerative agriculture, but is often based on heterodox political economic models that foreground the need for a radical redistribution of power in the food system. There is a risk that the mainstreaming, scaling up, and globalising of a singular model of regenerative agriculture will ride roughshod over the pre‐existing diversity of agro‐ecological practice. In its yet to be determined future, regenerative agriculture risks perpetuating colonial relations but also has the potential to learn from, integrate with, and enhance existing transformative practices. There is potential for important research tracing the current and future relationships between these two models.

There are parts of the world in which it makes more economic and environmental sense to raise livestock; situations in which lamb and beef might be a ‘benign extravagance’ (Fairlie, 2010). But in the context of continued demand for meat and the absence of concerted programmes for dietary shifts and demand reduction, there is a risk that the de‐intensification of livestock production in Global North – where the majority of carbon cowboys range – leads to a decline in yields that shifts the burden of food production to the Global South. This risks accelerating the large‐scale shift that is already underway in the global geographies of ecological restoration and ecological harm as agriculture comes to be concentrated in tropical regions. The abandonment of marginal land for rewilding projects and the shift to pasture‐fed systems that is underway in Europe and North America (Li & Li, 2017) is being enabled by the growth of often carbon‐intensive and biodiversity‐damaging plantation systems in tropical regions like Amazonia and Indonesia.

This is not to say that different actors in the countries implicated in the Southwards redistribution of the burden of food productivity are not experimenting with their own ruminant green rebranding. The agro‐ecology movement – which has a strong foothold in alternative, peasant farming foodscapes in the Global South – celebrates ruminants for many of the same reasons as regenerative farmers flagging their role in securing food sovereignty ambitions, and for the way their presence on the farm can help create the closed nutrient loops needed to end farmers' reliance on powerful agro‐chemical companies. Perhaps more relevant to the case study presented here are the global food businesses – including Danone, Nestlé, General Mills and McDonald's – who are reshaping their upstream global supply chains according to new regenerative principles (e.g., Danone, 2021). These initiatives are, however, all in their nascency and it is unclear which aspects of the regenerative model will be operationalised as it is made to touch down in landscapes in the Global South. It is also far from clear how successful these initiatives will be. As Freidberg (2017) notes, the bigness of ‘Big Food’ actors does not automatically furnish them with the knowledge or infrastructures needed to manage the sustainability of their own supply chains.

Irrespective of this traffic of ideas between the Global South and North, the rebranding of ruminants with reference to low‐intensity, extensive, regenerative practices risks reproducing colonial dynamics via the redistribution of intensive food production. It raises profound questions about the livelihoods of farmers in politically and economically marginal parts of the world, and environmental justice concerns as new and unequal geographies of exposure emerge as intensive farming shifts south. Research on the futures of regenerative agriculture will need to pay close attention to these issues, and to the ways in which farming and eating in different places are linked along globally connected supply chains.

Finally, this analysis speaks to several strands of the emerging discourse about the implications of the Covid‐19 pandemic for food systems. The pandemic has amplified the long‐standing concerns of epidemiologists and virologists that intensive, indoor livestock systems make zoonoses both more likely and more dangerous (Baudron & Liégeois, 2020; Wallace, 2016). Traditional, low‐intensity and extensive systems are regarded as less of an epidemiological risk (Brice et al., 2021). Although this has not yet been a prominent part of its allure, there are clear synergies between the anti‐intensive‐livestock sentiment prompted by Covid‐19 and the pastoral nature of regenerative livestock management. Relatedly, the pandemic has also been used as an opportunity to extol the merits of short supply chains and the resurgence of consumer–producer relations (Thilmany et al., 2021).

Although the changes in eating habits caused by lockdowns were highly uneven (Poelman et al., 2021) and it is far from clear how durable interest in alternative food systems might be, it is possible that the pandemic has created fertile conditions in which the regenerative green rebranding of ruminant animals might flourish further. These conditions might not, of course, be fully benign. The pandemic has generated its own share of defensive, xenophobic and nationalistic reactions (Sparke & Anguelov, 2020), and future research into the relationships between zoonoses and regenerative farming, alternative food movements and sustainable eating should pay attention to the diverse emerging politics of a post‐Covid society.

To conclude, this story of the green rebranding of cattle flags the need for new political temporalities for environmentalism in the Anthropocene; an era in which time is out of joint, and futures are arriving fast at speeds and in forms with no historical analogue. The past is an ever more foreign country, yet the environmentalist instinct to find solace in Nature dies hard. There remains a need to appreciate that appeals to a singular Nature are (and always have been) anachronistic. Nature is multiple, not a timeless and unmediated source of social and ecological value. And it therefore offers slippery grounds on which to base solutions. Nature offers any number of pasts and futures, but the process of deciding which to anticipate and affirm remains decidedly political.

## Data Availability

The research data are not shared.

## Endnotes

^1^
The term hooved locusts is commonly attributed to sheep, starting with John Muir and echoing over subsequent years, most recently in George Monbiot's (Monbiot, 2017) denigration of the English Lake District as a ‘sheep‐wrecked landscape’. In 1986, the American environmentalist Edward Abbey described the Western range as ‘cowburnt’ and denounced cattle as ‘hooved locusts’.

^2^
Ruminants are mammals that can obtain nutrients from cellulose‐rich plants by fermenting them before they are digested. This enables them to digest coarse material such as grass, husks and stalks which monogastric animals such as pigs, poultry and humans cannot. There are many kinds of farmed ruminants, including cattle, sheep and goats as well as minor species, such as llamas and camels. Cattle are by far the most important species as to numbers, impacts and food output (Garnett et al., 2017).
